# Uses of the Ophthalmoscope

**Published:** 1858-03

**Authors:** E. L. Holmes


THE CHICAGO

### MEDICAL JOURNAL.

VOL. I. MARCH, 1858. No. 3.

### ORIGINAL COMMUNICATIONS.

ARTICLE I.

### USES OF THE OPHTHALMOSCOPE.

BY E. L. HOLMES, M. D.

(READ BEFORE THE COOK CO. MEDICAL SOCIETY.)

Mr. President and Gentlemen,—In performing the duty you have assigned me this evening, allow me to ask your attention to a brief examination of the *Uses of the Ophthalmoscope.*

I need not dwell upon the importance and beauty of such an organ as the eye. To any inquiring mind the anatomy and physiology of this organ, and the investigation of the laws by which we explain the wonderful phenomena of light, offer one of the most interesting fields of labor in the domain of science.

There is scarcely an organ in the body which has more claim upon the interested attention of the pathologist than the eye, for not only can he observe the *effects* of disease, as witnessed after death, but he can trace with his own eye during the life of the sufferer the *processes* of disease, which in other organs are ever hidden from the view.

Every improvement in the means of diagnosis, by which we are better enabled to understand the manifold diseases which so often shroud the unfortunate in deepest darkness, must be welcomed by the pathologist, and much more by the practical physician, whose aim it is to prevent and cure such diseases.

The ophthalmoscope is comparatively of recent origin, and, as is the case with all new inventions, its merits have been thoroughly discussed by the most comprehensive minds of our profession.

It is certainly natural that any one, who has noticed how prone many have been to suggest new modes of treatment, to write books, to invent apparatus and improve upon instruments even of the simplest kind, who has visited some large museum of surgical and medical antiquities, and looked upon the infinite variety of appliances with the modifications they have received from physicians of every generation, should be disposed to question the utility of *any* invention, especially when he reflects how few of those of the past have stood the test of practical application.

But any one who has carefully examined the works of Graefe, Jaeger, Arlt and others, and who has had an opportunity to apply the ophthalmoscope himself, must be convinced of its great utility. A work upon the diseases of the eye, without a history and description of this instrument, as an aid to diagnosis, would be as incomplete as a work upon the diseases of the lungs without allusion to auscultation or percussion.

Before describing the ophthalmoscope, it would be perhaps well to consider for a moment why, in examining the posterior portions of the eye, the assistance of some such instrument becomes a necessity. We all well know it is impossible to trace the outline of any object in a room completely darkened, by placing the eye at a small aperture in the shutter, for the reason that all non-luminous bodies, to be seen, must receive light, in order that their images may be pictured upon the retina, by means of this light reflected from their surfaces. All that is necessary in examining an object in such a room is, by means of a simple reflector, to throw light either of the sun or from a strong burner upon the object and illuminate it, then, by placing the eye behind the reflector at a small opening through it, the examiner is able to see distinctly the object before him. Were the eye a simple hollow sphere, nothing could be done with less difficulty, than to examine the retina by means of light thrown through the pupil, as, by means of light, thrown through the shutter into the room just supposed. But such is not the case: the eye is filled with refracting media; the crystalline lens is a powerful magnifier, and an attempt to look through it by means of a simple reflector, would be very much like looking through a microscope without regard to its focal distances. Hence, it becomes absolutely necessary in examining the retina, to have an instrument, furnished with lenses, so arranged, that their focal distance may be adapted to the focal distance of the crystalline lens.

The principles upon which all ophthalmoscopes are constructed are similar, and as the instrument, originally proposed by Helmholz, has received a great number of modifications from Graefe, Jaeger, Reute, and, in fact, from nearly every oculist of celebrity, I will confine myself to the description of two varieties, one, by which we obtain an upright, and the other, by which we obtain an inverted image of the portion of the eye examined.

The former instrument consists of a plain or concave reflector, a couple of inches in diameter, surmounted upon a short straight handle. In the centre of this reflector is a small round aperture for the eye of the observer, immediately behind which opening is a small double concave lens. In examining an eye, it is necessary to sit directly in front of the patient in a darkened room, with a lamp giving a clear, steady flame, placed near the ear of the patient, so that the flame of the lamp, the eye of the patient, and the eye of the observer, shall fall in the same horizontal plane. By turning the reflector to a proper angle with the rays from the lamp, the light is thrown through the pupil, and by moving the instrument to and from the eye under examination, the requisite focal distance is found, when everything behind the crystalline lens may be seen magnified about twenty-four times.

The other instrument consists of a concave *reflector*, as in the former variety, without the concave *lens* behind the aperture in the centre; but in its stead, a strong double convex glass is held very near the eye of the patient, in such a manner that the light from the lamp is reflected through this lens before entering the eye. This lens must of course be moved backwards and forwards till a distinct image of the portion of the eye examined is obtained. Examined in this way, the different portions of the retina are magnified four or five times. But the image of the retina is *inverted;* that is to say, the vessels of the upper portion of the retina appear as if in the lower portion, and *vice versa.*

An eye when illuminated by either of these methods without any reference to focal distances presents a clear, light yellowish disk, equal in size to the pupil. When, however, the lens is properly adjusted, a well-defined and beautiful picture of the retina, with its vessels and the entrance of the optic nerve, is obtained. The ground color of this picture is a beautiful yellowish red. The optic papilla, however, situated, as is well known, not directly opposite the pupil, but towards the nasal side of the eye, is seen as a well-defined disk of a fine light orange tint. The central artery and vein send their branches principally to the upper and lower portions of the retina, leaving the lateral portions almost destitute of vessels. It should be remembered, that in the human eye unmagnified, the entrance of the optic nerve is not more than a line or a line and a half in diameter; the veins and arteries are exceedingly minute, the former being somewhat larger and darker than the other.

A perfect representation of the posterior portion of the concave surface of the eye is most faithfully delineated in the fine work of Jaeger, *Beitraege zur Pathologie des Auges.*

The cause of the peculiar red color of the eye, as viewed by the ophthalmoscope, has been the matter of some discussion among the best authorities. The probability is that it depends almost entirely upon the pigment layer of the choroid, and not upon the quality of the light employed, nor upon the blood circulating in the substance of the retina.*

* The color of the posterior concavity of the eye in the *white races,* according to all authorities, is as above described.

My friend Dr. J. B. Lyman, of this city, has examined the eyes of two

To obtain a distinct view of the retina requires, on the part of most beginners, considerable practice; for not only does the cornea, being a good reflector, give a brilliant image of the lamp, which much disturbs the vision of the inexperienced, but the crystalline lens of the patient, in consequence of the delicate organization by which it accomodates itself to near or distant objects, is, with some persons, even when directed to fix their eyes upon a particular object, constantly throwing the portions of the eye behind it out of focus. The examiner is, therefore, obliged to move his ophthalmoscope either to or from the eye, as the case may require, to keep it in focus, a thing by no means so easily accomplished as one might at first imagine.

Before entering upon the details of the practical application of the ophthalmoscope in the diagnosis of disease, a few words need to be said regarding the cornea in certain abnormal conditions, and regarding the mode by which we locate the different diseases which come under our observation. It is true in diseases of this portion of the eye, the assistance of the ophthalmoscope is seldom required, for the simple reason that they are external and easily observed, still in one or two forms of disease the unaided eye is often scarcely able to determine their extent, which at once become very evident with the help of the ophthalmoscope. But this is not the principal point to which I wish to draw your attention.

One can readily perceive that any cicatrix or macula upon the cornea in front of the pupil, or upon either of the two convex surfaces of the crystalline lens, when any attempt is made to examine the deeper portions of the eye with the ophthalmoscope, must necessarily cast a shadow *upon* these portions, and it is sometimes difficult to decide which membrane is diseased unless we take advantage of a principle well known to astronomers in calculating the parallax of the planets.

Suppose for an instant, an obscuration upon the cornea, another upon the anterior, and a third upon the posterior surface of the crystalline lens, each one being situated directly in the antero-posterior axis of the eye. It is evident if the

---

healthy negroes, and states that he found the color to be nearly black, thus showing the same development of pigment in the choroid coat as in the cutis.

eye of the observer be placed directly in a line with these points, they will appear as one obscuration; just as three lamps placed in a line with the axis of the eye appears like a single flame.

If, however, the observer moves his eye sideways, in either direction, so as to view the eye obliquely, the relative situation of these supposed obscurations will be changed, and one can decide with perfect confidence where the disease is situated. In viewing the spot upon the cornea from one side, it will cast its shadow upon the edge of the iris, situated upon the side opposite to the direction in which the observer moved. The spot upon the anterior surface of the lens, inasmuch as it practically lies not *behind* the iris, but in the very centre of the pupil, will always *appear* in the center of the pupil, let the observer view it from any direction he may choose. But the spot upon the posterior surface of the lens, as well as any object lying behind it, will appear to fall on moving the eye in either lateral direction, in a line with that portion of the iris lying on the same side to which the observer moves his eye.

The diseases of the refracting media of the eye, lying behind the iris, offer a most interesting though somewhat limited field for the application of the ophthalmoscope. From the fact that light thrown directly into the eye will usually contract the pupil, it is well, though by no means always necessary, to apply a few drops of the solution of atropine upon the conjunctiva, a few moments before the examination is commenced, simply to counteract the influence of the light.

The diseases of the lens are almost all included in the different species of cataract. We all well know that the subjective symptoms are peculiar, and that the practitioner is enabled with the unaided eye to diagnose the disease in its advanced stage, yet very frequently long before the patient suspects any change, or the physician can detect any obscuration with the usual means of diagnosis, the ophthalmoscope reveals the slowly forming cloud which is to shut out the precious light of heaven from the unfortunate sufferer. This is certainly a source of satisfaction to the practitioner; moreover, we should remember it not unfrequently happens, that synchronous with the origin of the *lenticular* disease, another abnormal process is going on in the *retina* or *choroid*. The ophthalmoscope discloses the true condition of the different portions of the eye, shows the physician that the increasing loss of vision is not wholly due to the cataract, and also assists him in giving an enlightened and rational opinion regarding the probable result of a future operation. The appearance of the cataract, as seen by the ophthalmoscope, is often exceedingly beautiful. A description of the different varieties would be useless, since a glance at the plates Nos. II. and III. of Jaeger's larger work (*Beitraege*), and No. III. of the smaller one (*Staar und Staar Operationen*), will give a far better idea than any description.

The vitreous humor is liable to several abnormal conditions, which it is not only interesting but instructive to examine with the accuracy with which we view an object in the field of a microscope. The infiltration of blood in its various forms, the deposition of pigment cells, which are found not only fixed in different portions of the humor, but floating within it at every motion of the eye, the rare and singular appearance of cholesterine crystals, which are occasionally developed in the vitreous humors,—are all most beautifully opened to our vision by means of the instrument before us. Graefe, of Berlin, and others, report several cases of cysticercus in the vitreous humor of the human eye. Without the assistance of the ophthalmoscope, it would be quite impossible to give a diagnosis, from the simple subjective symptoms of the patient. With its aid we are able to trace the extent, form, color and mobility of all the objects just mentioned, and also to witness the motions of the singular entozoon, which sometimes so strangely finds its abode within the eye.

We now come to a numerous class of diseases, which claim the most careful attention of the practical oculist—the diseases of the retina and choroid, among the most grave to which the eye is subject.

Before examining the abnormal appearances produced by these diseases, allow me to direct your attention for a moment to the anatomy of the retina and choroid coat.

The retina, consisting of several lamina, is a transparent membrane, varying in apparent color, according to the amount of blood it contains, or of the coloring matter deposited under it, is the first portion of the eye we observe behind the vitreous humor. Next in order is the pigmentum of the choroid, which consists of a layer of hexagonal cells, containing, according to the peculiarities of individuals, a greater or less amount of pigment. The other two lamina of the choroid are composed of a stroma, in which pass very freely the numerous veins and arteries, the most important, perhaps, of the eye; to the former of which, from their peculiar conformation, is given the term, "venæ vorticosæ." The stroma of this layer is also provided with pigment cells, though in much less quantity than in the true pigment *layer*.

These facts being borne in mind, we shall be better able to understand the diseases we are about to examine.

Probably, the most frequent affection of the retina which we are able to detect by means of the ophthalmoscope, and which is found more or less among all classes of people, who employ their eyes to any extent upon small and near objects, is a slight congestion—"hyperamæa retinæ," as termed by Jaeger. This state of the retina is characterized particularly by a slight diminution of distinctness in the line of demarkation, between the circumference of the optic papilla and the surrounding portions of the retina, which, in perfect health, is like the papilla of plate I. of Jaeger's larger work. By comparing this plate with plates V. and IX., the changes to which I refer will be readily seen. If the congestion passes into the state of inflammation, we shall find the optic nerve almost wholly, if not entirely, obscured, and from it will be seen radiating innumerable, delicate, ill-defined lines, and many times a thin film will appear to rest upon the surface of the diseased retina. While these changes are going on, others, if possible, of a graver nature, often manifest themselves. Lymph is deposited either *upon* or under the retina, destroying its delicate texture, covering in many places the vessels; in fact, rendering them almost invisible. Under such circumstances, vision is many times wholly destroyed. These changes are beautifully illustrated in the last three plates of Jaeger. I would particularly call your attention to plate No. XIII., representing a large deposit of lymph.

The disease known as dropsy of the choroid or hydrops sub-retina, in which the retina becomes separated from the choroid and floats upon the fluid beneath, although, in some cases, easily recognized by the unassisted eye, is nevertheless far better seen by the ophthalmoscope. The extent of the lesion of course varies. Occasionally, it embraces the whole lower hemisphere of the eye. Every motion of the eye throws this floating membrane into waves, which pass from one side of the globe to the other. Usually, the blood-vessels of this portion of the retina become atrophied, and finally appear as dark lines, as shown in plate IV., fig. XXIV., of Jaeger's smaller work.

From what we have already seen, there is no need of argument to demonstrate the utility of the ophthalmoscope in some of the rarer forms of disease; as, for instance, apoplexy of the retina, aneurism of the central artery and its branches, ossification or laceration of the retina.

Before leaving the retina, I wish to say a few words regarding a beautiful and singular *phenomenon*—the pulsation of the central artery and vein. This is an interesting and important subject, on account of its connection with the disease commonly known as glaucoma. It is not my purpose to enter upon any discussion regarding the nature of this disease, but simply to state that, according to those ophthalmologists, to whose accuracy of observation, acquired in a rich field of practical experience, we can most trust, the ophthalmoscope has revealed two new diagnostic signs—the pulsation of the vessels to which I have just alluded, and a peculiar prominence or projection forward of the optic papula, caused probably by a deposition of a yellowish blue and sometimes red exudation upon the central portion of the papilla, leaving but a narrow circle of the natural structure around it. The first mentioned symptom is of considerable importance, and seems to depend upon an unnatural fulness of the eye, causing an abnormal *pressure* upon the vessels, for, it is found, one can readily produce the same phenomenon of pulsation in the central artery and vein of animals by pressing somewhat forcibly with one or more fingers upon the eye. In an eye affected with glaucoma in an advanced stage, this pulsation is spontaneous and constant; while, in the commencing stages, a *slight* pressure only is required from the finger to cause the artery and vein to pulsate. In health, the pressure of the contents of the eye outward is of course normal, and no pulsation is visible; but, in glaucoma, this pressure seems to be too great, from an *increase* in the quantity of fluids. Why pressure either from without, or from an increase of fluids within, should cause this pulsation, is still a subject of doubt. I am sorry I have no plate by which I can illustrate to you the peculiar appearance caused by deposition of lymph upon the optic papilla.

The choroid coat of the eye is the seat of numerous important affections. Graefe reports that out of a thousand cases of amblyopia or incipient amaurosis, 425 showed evident signs of disease in this membrane. Thus far we have examined those portions of the eye which are more or less transparent, and if the pigment coat of the choroid always remained in a normal condition, it would limit our researches with the ophthalmoscope. But the pigment layer itself is subject to disease. For some reason not altogether easy to explain, this lamina becomes absorbed, leaving the portions beneath perfectly visible. If we cannot explain this process satisfactorily, it may perhaps not appear quite so strange that the eye should be affected in this way, when we recollect how frequently, under certain circumstances, pigment cells are no longer deposited at the follicles of the hair, which therefore soon becomes grey. As regards the choroid, there seems often to be a *double* abnormal process in the *same* eye. For while we find in some portions of an eye the pigment absorbed faster than it is deposited, leaving, as I have just stated, the layers under it visible, in other portions the pigment is deposited faster than it is absorbed; here, of course, the pigment is found in larger quantities than normal, sometimes seeming to involve the *retina* and its vessels. It should be remarked, however, that these dark deposits are believed by some, and doubtless many times with reason, to be the coloring matter of extravasated blood.

The aspect of the posterior portion of the eye, after the pigmentum has been absorbed, is very peculiar. The vessels of the choroid are seen passing in graceful loops, anastomosing freely with each other, *beneath* the vein and artery of the retina, like very narrow bands, which, unlike the vessels of the retina, retain nearly the same width throughout their entire extent. The color of these vessels depends upon the amount of coloring matter yet remaining over them. Where the true pigment layer is wholly absorbed, their color is of a decided yellow cast; those which lie deeper in the stroma of the choroid, and are consequently covered with a certain amount of pigment, have a darker color. A fine example of these appearances is given in Jaeger's work, *plate VI.* Plate IX. of the same work gives a good idea of similar abnormal conditions, somewhat circumscribed in extent.

There remains but a single point to which I ask your consideration, the use of the ophthalmoscope in the diagnosis of staphyloma posticum. You are well aware that the cornea is liable to a diseased action, in consequence of which, it is rendered insufficient to resist the normal pressure of the contents of the eye, and ordinary staphyloma is the result. A similar process sometimes has its seat in the posterior portion of the membranous tunics of the eye, and they project posteriorly, as does the cornea anteriorly. This state of things is accompanied with a great degree of myopia, and according to Jaeger, where near-sightedness is *extreme*, approaching perhaps to amaurosis, staphyloma posticum to a greater or less degree is in numerous instances an accompanying evil. The probability, however, is, that the staphyloma increases the distance between the retina and crystalline lens. In order that the rays from an object in front of the eye may be brought to a focus on the retina, in the disease we are considering, they must be rendered less converging, which, of course, is only accomplished, when a lens is not employed, by bringing the object nearer the eye.

The peculiar appearance, considered by many as pathognomonic of the disease in its early state of development, is a change which occurs around the external portion of the optic papula. I have no colored plate by which I can exhibit the eye as it appears in this disease. The color of the affected portion is similar to that of the papula itself; its form resembles that of a crescent, the concavity being placed in juxtaposition with the outer semi-circumference of the papula; sometimes the convexity of the crescent-shaped figure is elongated into a kind of ellipse.

Several theories have been devised to explain the origin, form and color of this pathological growth, but none of them seem without objection. The subject is a new one, and the relation between staphyloma posticum and the appearance just described, is still open to the investigation of the pathologist and practical physician.

Gentlemen, I have finished what I have to say upon the practical use of the ophthalmoscope. I am well aware how meagre and superficial my remarks have been. To discuss all the principles upon which the instrument is constructed, all the questions which naturally arise regarding the anatomy, physiology and pathology of the human eye, could by no means be accomplished within the limits of a single paper. I have, therefore, confined myself to a simple, practical statement of what the ophthalmoscope is capable. The field is a comparatively new one for the scientific observer, and there remains for him many obscure problems to solve.

An objection has been raised against the use of the ophthalmoscope, founded, however upon insufficient grounds. It is stated that light reflected so directly into an eye, especially if diseased, must exert a deleterious influence. Doubtless, in cases of severe inflammation, especially with marked photophobia, it would be imprudent to attempt to examine the eye with artificial light, and no wise practitioner would think of doing it, for the simple reason, that in such cases the ophthalmoscope could render little aid in giving a diagnosis. In all cases, in which the patient can without inconvenience bear the light of a common lamp held but a couple of feet from his eyes, no evil can follow. From what I have seen, in a large number of patients under the care of others, and in my own experience, I am convinced that it very rarely happens that a patient experiences either pain or inconvenience when the above precaution is observed.

The ophthalmoscope is still imperfect. We need an instrument with a magnifying power sufficiently great to enable us to detect and examine with greater accuracy the pathological changes which we have briefly considered; for, it is found by experience, that the *subjective* symptoms do not always correspond to the objective symptoms as revealed by the ophthalmoscope. As, for instance, we may observe an apparently grave degree of congestion of the retina, or a partial absorption of the pigmentum, or a deposition of lymph, and yet the patient may suffer much less from troubled vision than another with comparatively few visible signs of disease.

Here is wide scope for the talent and acumen of the true observer. To detect symptoms is certainly one of the most important parts of the physician's duty; but it is a no less important duty to interpret correctly the symptoms as they appear, and in many cases of diseases of the eye, especially in those in which the ophthalmoscope is of any assistance to the physician, this is one of the most difficult tasks we have to perform.

As we look back, however, upon the past six or seven years, and consider the number of facts, interesting and important, which have been discovered, and the doubtful points that have been explained away, particularly by the German ophthalmologists, we can confidently look forward to still greater results in the future.

I can now only add—I thank you for your attention, and as regards this brief sketch of the subject, I trust it has not been altogether void of interest to you, and that, if it has contributed no novel truths, it has at least shown that the subject is worthy of the careful investigation of the practical and scientific observer.

